# *MET* nucleotide variations and amplification in advanced ovarian cancer: characteristics and outcomes with c-Met inhibitors

**DOI:** 10.18632/oncoscience.3

**Published:** 2013-12-11

**Authors:** Chad Tang, Denis L. Fontes Jardim, Gerald S. Falchook, Kenneth Hess, Siqing Fu, Jennifer J. Wheler, Ralph G. Zinner, Aung Naing, Apostolia M. Tsimberidou, Debora De Melo Galgiato, Shannon N. Westin, Funda Meric-Bernstam, Razelle Kurzrock, David S. Hong

**Affiliations:** ^1^ Departments of Radiation Oncology, The University of Texas MD Anderson Cancer Center, Houston, TX.; ^2^ Department of Investigational Cancer Therapeutics, The University of Texas MD Anderson Cancer Center, Houston, TX.; ^3^ Department of Gynecology Oncology, The University of Texas MD Anderson Cancer Center, Houston, TX.; ^4^ Department of Biostatistics, The University of Texas MD Anderson Cancer Center, Houston, TX.; ^5^ Moores Cancer Center, University of California San Diego Health System, San Diego, CA.

**Keywords:** MET nucleotide variations, MET amplification, ovarian cancer, c-Met inhibitor

## Abstract

**Purpose:**

*MET* alterations including amplifications and nucleotide variations have been associated with resistance to therapy and aggressive clinical behavior.

**Experimental Design:**

The medical records of patients presenting to the University of Texas MD Anderson Cancer Center Phase I Clinic with relapsed or metastatic ovarian cancers and known *MET* nucleotide variation or amplification status were reviewed retrospectively (n=178). Categorical and continuous clinical and molecular characteristics were compared using Fisher's exact and Wilcoxon rank-sum tests, respectively. Univariate and multivariate survival were assessed via Kaplan-Meier and Cox regression analysis, respectively.

**Results:**

*MET* amplification occurred in 4 (3.5%) of 113 patients, whereas nonsynonomous nucleotide variations were present in 9 (7.4%) of 122 patients. No patients exhibited concomitant amplification and variation. *MET* variations were observed only in white women with high-grade ovarian tumors, whereas amplifications were observed in both black and white women with high-grade serous ovarian primary tumors. No patients (n=4) exhibiting a *MET* alteration achieved an objective response when treated on a c-Met inhibitor phase I trial. In addition, ovarian cancer patients treated with a c-Met inhibitor with multikinase activity trended towards a longer time-to-failure compared with those treated with a c-Met-specific inhibitor (median: 1.5 vs. 4.5 months, p=0.07).

**Conclusions:**

*MET* alterations occur in a minority of patients with ovarian cancer. c-Met inhibitors with multikinase activity may exhibit less activity in ovarian cancer than c-Met specific drugs. These findings warrant further investigation.

## INTRODUCTION

The c-Met tyrosine kinase receptor (TKR), upon activation by its cognate antigen, hepatocyte growth factor (HGF), generates proliferation, migration, and survival signals in numerous cancers[1, 2]. This signaling cascade parallels, and at times can supplement, the activity of other oncogenic TKRs. As a result, the c-Met pathway has emerged as a resistance pathway in therapies targeting the epidermal growth factor receptor (EGFR)[3, 4], B-Raf[5], and vascular endothelial growth factor receptor (VEGFR)[6], among others. Various *MET* alterations, including amplification and nucleotide variations, have been described and are associated with resistance to therapy and aggressive clinical behavior[3, 4, 7]. The pathologic implications of this important receptor has prompted the development of c-Met inhibitors, many of which are currently undergoing early phase trials in various cancers[2].

Ovarian, primary peritoneal, and fallopian tube cancers have similar ontological origins and clinical presentations. Their aggressive metastatic behavior and generally poor prognosis has prompted interest in developing therapies with TKR inhibitors, including those targeting EGFR and c-Met[8, 9]. However, outside of bevacizumab, targeted therapies tested in early clinical trials have yet to gain widespread clinical success[8]. Despite understanding of the intrinsic biology of the c-Met pathway and its documented role in drug resistance, no substantive clinical series have assessed the effect of *MET* variations and amplifications in this disease[10-12]. We, therefore, investigated the clinical and molecular characteristics of patients with ovarian cancers referred to our Phase I Clinical Trials Program and their response to treatment on a phase I c-Met inhibitor trial.

## RESULTS

### Patient Characteristics

One-hundred-and-seventy-eight patients met study inclusion criteria, of whom 122 and 113 were tested for *MET* variations and amplification, respectively. Fifty-seven patients were tested for both variation and amplification. *MET* nucleotide variations were detected in 9 patients (7.4%): 6 with N375S and 3 with T1010I nonsynonomous variations. *MET* amplification was detected in 4 patients (3.5%), amplification gene copy numbers (in relation to *CEP7*) were 2.12, 2.27, 2.55, and 2.78. No patients exhibited concomitant *MET* nucleotide variation and amplification.

### Characteristics associated with *MET* aberrations

No significant differences were noted between patient characteristics when stratified by *MET* variation or amplification status (all p>0.05). *MET* variations were detected only in white women with high-grade primary ovarian tumors (Tables 1 and 2). The histology of cancers with *MET* variations was predominately serous (74%), with one patient each having carcinosarcoma and clear cell carcinoma (Table 1). The median numbers of metastatic sites among patients with *MET* variations compared with those without were both 2. Among patients with *MET* variations, 44% had liver metastasis, a rate similar to liver metastasis in patients without *MET* variation (34%). Concomitant mutations included *KRAS*, *BRCA1*, *ARID1A,* and *TP53* were identified in 1, 1, 1, and 2 patients, respectively. In addition, one patient had concomitant PTEN loss and another had weak PTEN staining (Table 2). Of note, no ovarian cancer patients within any stratum exhibited *ALK* rearrangement, *BRAF*, *EGFR*, or *KIT* mutations.

**Table 1:** Demographic characteristics and metastatic sites in patients stratified by MET nucleotide variation and amplification status

Characteristic	Wild-Type (n=113)	Variation (n=9)	Not amplified (n=109)	Amplified (n=4)
Age Diagnosis: Median (Q3-Q1)	54 (47-61)	54 (46-56)	54 (47-61)	60 (54-67)
Prior Therapies: Median (Q3-Q1)	3 (2-5)	3 (2-5)	3 (2-5)	2 (1-5)
Diagnosis				
Fallopian	11 (9%)	0	1 (1%)	0
Peritoneal vs. Ovarian	2 (2%)	0	1 (1%)	0
Peritoneal	5 (4%)	0	2 (2%)	0
Ovarian	95 (84%)	9 (100%)	105 (96%)	4 (100%)
Ethnicity				
Asian/AM-indian	2 (2%)	0	2 (2%)	0
Black	3 (3%)	0	4 (4%)	1 (25%)
Hispanic	11 (10%)	0	9 (8%)	0
White	97 (86%)	9 (100%)	94 (86%)	3 (75%)
Histology				
Serous	75 (66%)	7 (78%)	70 (64%)	4 (100%)
Endometrioid	5 (4%)	0	5 (5%)	0
Clear Cell	9 (8%)	1 (11%)	13 (12%)	0
Mucinous	0	0	2 (2%)	0
Mixed	11 (10%)	0	13 (12%)	0
Carcinoma NOS	2 (2%)	0	1 (1%)	0
Carcinosarcoma	5 (4%)	1 (11%)	1 (1%)	0
Granulosa	6 (5%)	0	4 (4%)	0
Metastasis				
# Met Sites: Median (Q3-Q1)	2 (2-3)	2 (2-4)	2 (2-3)	2 (2-3)
Liver	39 (34%)	4 (44%)	48 (44%)	1 (25%)
Lungs	23 (20%)	3 (33%)	20 (18%)	0
Bone	6 (5%)	0	7 (6%)	0
CNS	4 (4%)	0	4 (4%)	0
Peritoneum	102 (90%)	8 (89%)	97 (89%)	4 (100%)
Other Sites	83 (73%)	7 (78%)	81 (74%)	3 (75%)

Abbreviations: (Q3-Q1) = 75th to 25th percentile, AM-indian = American indian, NOS = not otherwise specified, Met = metastatic, CNS = central nervous system.

**Table 2:** Histologic and genetic characteristics in patients stratified by MET nucleotide variation and amplification status

Characteristic	No Variation (n=113)	Variation (n=9)	Not amplified (n=109)	Amplified (n=4)
HER2 amplification	4/73 (5%)	0/5	3/71 (4%)	0/2
ALK rearrangement	0/36	0/2	0/38	0/1
PIK3CA mutation	8/109 (7%)	0/9	10/104 (10%)	0/4
KRAS mutation	11/105 (10%)	1/8 (13%)	7/90 (8%)	0/3
EGFR mutation	0/86	0/4	0/76	0/3
TP53 mutation*	34/69 (49%)	3/4 (75%)	18/33 (5%)	0/1
BRAF mutation	0/103	0/8	0/82	0/3
NRAS mutation	2/96 (2%)	0/3	0/45	0/2
KIT mutation	0/83	0/3	0/48	0/1
% ER+: median (Q3-Q1)	70 (20-90) N=86	30 (20-80) N=7	70 (5-90) N=81	35 (13-65) N=4
% PR+: median (Q3-Q1)	10 (5-30) N=50	N=0	5 (1-38) N=28	3 (1-5) N=2
PTEN				
Loss	7/77 (9%)	1/8 (13%)	4/81 (5%)	2/4 (50%)
Weak	10/77 (13%)	1/8 (13%)	15/81 (19%)	0/4
No Loss	60/77 (78%)	6/8 (75%)	62/81 (77%)	2/4 (50%)
Grade				
Low	9/97 (9%)	0	6/87 (7%)	0
Medium	2/97 (2%)	0	2/87 (2%)	0
High	86/97 (89%)	6/6 (100%)	79/87 (91%)	4/4 (100%)
Heredity				
1st Degree	33/113 (29%)	2/9 (22%)	30/109 (28%)	1/4 (25%)
2nd Degree	40/113 (35%)	3/9 (33%)	33/109 (30%)	2/4 (50%)
BRCA1 or 2 mutation	10/31 (32%)	1/2 (50%)	9/28 (32%)	0/2

Numerator within each cell indicates number of patients exhibiting the characteristic while denominator indicates number of patients tested

Abbreviations: (Q3-Q1) = 75th to 25th percentile, % ER+ = percent of cell staining positive for estrogen receptor, % PR+ = percent cells staining positive for progesterone receptor, 1st Degree = presence of any first degree relative with either breast or ovarian cancer, 2nd Degree = presence of any second degree relative with either breast or ovarian cancer

* TP53 testing was conducted by hotspot analysis

*MET* amplification occurred only in women with high grade serous ovarian cancer (Tables 1 and 2). Three out of 4 women were white and 1 was black. Similar to patients with *MET* variations, *MET* amplified patients had a median of 2 metastatic sites, with 1 patient exhibiting liver metastasis. No concomitant mutations were observed in *MET* amplified patients except for in 2 patients who exhibited loss of PTEN protein expression.

### *MET* alterations and survival

**Figure 1:**
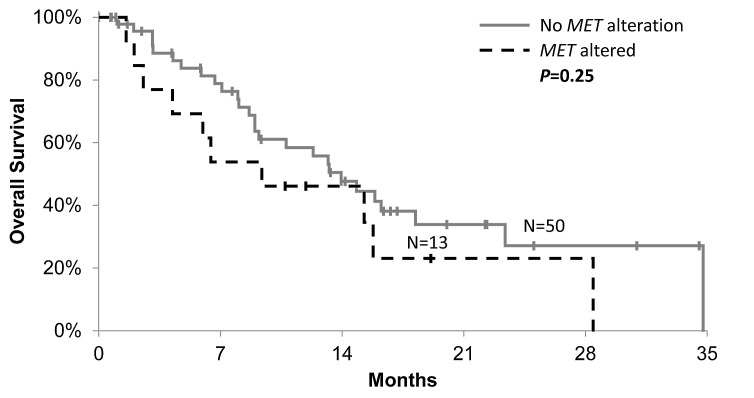
Kaplan-Meier plot of overall survival in ovarian cancer patients with MET variation or amplification (dashed-black line) compared with patients without MET variation or amplification (solid-gray line)

OS in patients with a *MET* aberration (either variation or amplification, n=13) was compared with that of patients known to be negative for any aberrations (n=50). There was no significant different in median survival in patients exhibiting *MET* alterations (Fig. 1; HR=1.6, p=0.25). Patients with *MET* alterations trended towards worse OS in multivariate analysis (HR=1.8, p=0.13) when adjusting for histology (serous vs. other), age (≥60 vs. <60), and number of prior therapies (>3 vs. ≤3).

### Treatment with c-Met inhibitors

All patients treated on a Phase I c-Met inhibitor clinical trial had a diagnosis of ovarian cancer. This included 4 patients with *MET* nucleotide variation and one with *MET* amplification (Table 3). No patients with a *MET* alteration achieved an objective response.

**Table 3:** Histology and mutation status of patients exhibiting MET variation or amplification, and their response to c-Met inhibitors

Patient No.	Histology	Variation/Copy Number	Concomitant Mutations	Inhibitor Class	Best Response	TTF (mo)
MET variation						
1	Serous	T1010I	BRCA1	c-Met specific	PD (+29)	1
2	Serous	T1010I	-		-	-
3	Serous	T1010I	TP53		-	-
4	Carcinosarcoma	N375S	-	c-Met specific	PD (+39)	1.2
5	Clear Cell	N375S	PTEN loss	Multikinase	SD (+19)	1.2
6	Serous	N375S	KRAS	Multikinase	SD (+16)	1.5
7	Serous	N375S	TP53		-	-
8	Serous	N375S	-		-	-
9	Serous	N375S	TP53, ARID1A		-	-
MET amplification						
10	Serous	2.12	PTEN loss	Multikinase	PD(*)	2.1
11	Serous	2.27	PTEN loss		-	-
12	Serous	2.55	-		-	-
13	Serous	2.78	-		-	-

Abbreviations: PD = progressive disease, SD = stable disease, TTF = time to failure

* Indicates clinical progressive disease

**Figure 2:**
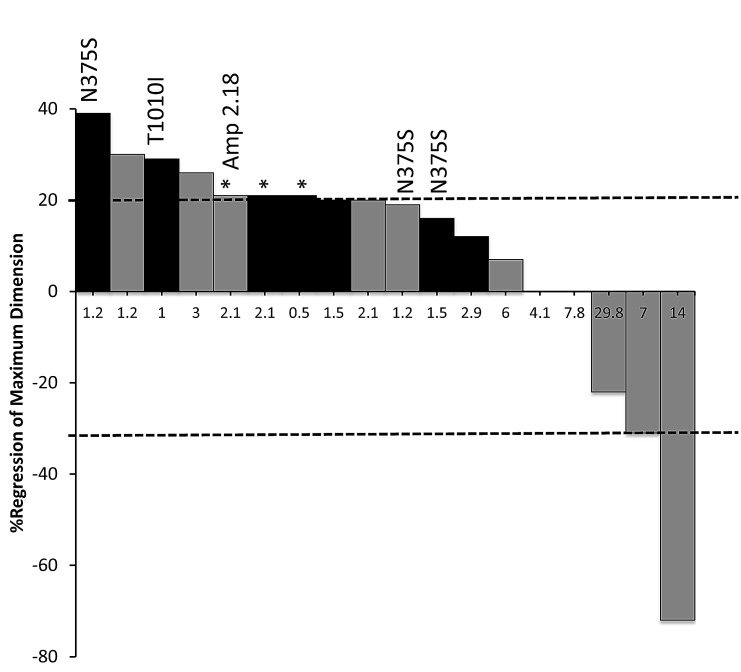
Waterfall graph of ovarian cancer patients treated on a c-Met inhibitor Phase I trial with measurable disease by RECIST criteria Patients exhibiting *MET* alteration are annotated above the bar with the corresponding variation or fold-amplification (in relation to *CEP7*). Patients treated with a c-Met inhibitor with multikinase activity are displayed with grey bars, while those treated with a c-Met specific inhibitor are displayed with black bars. X-axis indicates time-to-failure (months). (*) Indicates clinically progressive disease.

Among all patients with ovarian cancer treated on a c-Met inhibitor trial, 5 out of 18 (28%) exhibited a partial response (PR) or stable disease (SD) lasting ≥6 months. Of these, 2 of 18 patients (11%) achieved a best response of PR (Fig. 2, Table 4). Prolonged SD lasting ≥6 months was achieved in 3 patients with TTFs lasting 6, 7.8, and 29.8 months. The median TTF in patients treated with a c-Met-specific inhibitor (1.5 months, range 0.4-7.8 months, n=9) was less than patients treated with a multikinase inhibitor (n=8) or a multikinase inhibitor combined with a VEGFR2 inhibitor (n=1)(4.5 months, range 1.2-29.8 months). This difference trended towards significance (p=0.07). Interestingly, among 3 patients with known *TP53* mutations treated on a c-Met inhibitor trial, the 2 patients treated with a c-Met inhibitor with multikinase activity exhibited objective responses, while the one patient who was treated with a c-Met specific inhibitor did not (Table 4).

**Table 4:** Histology, mutation status, and response of ovarian cancer patients treated on a phase I c-Met inhibitor trial

Patient No.	Histology	MET Variation/Amp	Other Mutations	Inhibitor Class	Best Response	TTF (mo)
1	Serous	Amp-	TP53	Multikinase	PR (−72)	14
2	Endometroid	Var−/Amp−	PI3KCA, STK11	Multikinase+VEGFR2	PR (−31)	7**
3	Serous	Var−/Amp−	TP53	Multikinase	SD (−22)	29.8
4	Clear Cell	Var−	-	c-Met specific	SD (0)	4.1
5	Clear Cell	Amp−	HER2 amp	c-Met specific	SD (0)	7.8
6	Serous	Amp−	-	Multikinase	SD (+7)	6
7	Serous	Var−/Amp−	KRAS, TP53	c-Met specific	SD (+12)	2.9
8	Serous	N375S/Amp−	KRAS	c-Met specific	SD (+16)	1.5
9	Clear Cell	N375S/Amp−	PTEN loss	Multikinase	SD (+19)	1.2
10	Serous	Amp−	-	c-Met specific	PD (+20)	1.5
11	Mixed: Serous/Transitional	Amp−	PI3KCA	Multikinase	PD (+20)	2.1
12	Mixed: Serous/Epithelial	Amp−	-	c-Met specific	PD (*)	2.1
13	Serous	2.18	PTEN loss	Multikinase	PD (*)	2.1
14	Serous	Var−	-	c-Met specific	PD (*)	0.5
15	Serous	Amp−	-	Multikinase	PD (+26)	3
16	Serous	T1010I/Amp−	BRCA1	c-Met specific	PD (+29)	1.0
17	Serous	Amp−	-	Multikinase	PD (+30)	1.2
18	Carcinosarcoma	N375S	-	c-Met specific	PD (+39)	1.2

Abbreviations: AMP = amplified, Amp− = tested, no MET amplification, Var = variation, Var− = tested, no MET nucleotide variation, PD = progressive disease, SD= stable disease, amp=amplification, TTF=time-to-failure

* Indicates clinical progressive disease

** Indicates continuation on a c-Met trial

## DISCUSSION

The literature delineating the prevalence of *MET* amplification and variations in ovarian cancer is sparse. Yamato et al. reported on 5 patients, all with clear cell histology, identified with *MET* amplification out of 195 (2.5%) patients tested[13]. An earlier report using Southern blot analysis in 67 patients with ovarian cancer found no amplifications[12]. Two other series with 24 and 65 ovarian cancer patients found the incidence of *MET* variations to be 1 and 0, respectively [10, 11]. The rates in our current cohort (nucleotide variations, 7.4%; and amplification, 3.5%) are comparable, albeit higher, than those reported in previous studies. A possible explanation is that our study population consisted entirely of patients with metastatic or relapsed disease, which may skew in favor of increased rates of *MET* aberrations. Of note, we did not detect any patients with concomitant *MET* amplification and variation.

The biologic activity of the *MET* nucleotide variations identified here and even whether these specific variations represent somatic mutations or germline polymorphisms have yet to be fully elucidated[17, 18]. The role of *MET* amplification has been most extensively correlated with tumor invasion and aggressive metastatic behavior in gastrointestinal malignancies[19, 20]. With regard to *MET* variations, the N375S nonsynonmous variation occurs in the extracellular semaphorin domain, whereas the activating T1010I variation occurs in the juxtamembrane domain[7, 18]. The characteristics and clinical behavior of ovarian cancer in patients with MET variations have not been thoroughly described. However, data in renal cell and lung carcinomas suggest that somatic and germline *MET* polymorphisms may enhance c-Met TKR activity and even confer inhibitor resistance[17, 21]. This second observation is corroborated by our data, in which no patients exhibiting a *MET* alteration achieved an objective response on a c-Met phase I inhibitor trial. Another possible explanation for the lack of an observed objective response are that most patients in this study were enrolled on a Phase I dose escalation trials and may not have received an efficacious study drug dose. Few preclinical data have tested the efficacy of these inhibitors in *MET* altered patients [22-24] and to our knowledge no associated clinical data have been reported.

A subset of all ovarian cancer patients achieved objective responses. Patients treated with a multikinase inhibitor trended toward a longer TTF compared with those treated with c-Met-specific inhibitors (median 1.5 vs. 4.5 months, p=0.07). A possible explanation is that activity against VEGFR2, a target with known clinical efficacy in ovarian cancer[14], may increase therapeutic efficacy. Interestingly 2 out of 3 patients exhibiting *TP53* mutations exhibited objective responses to c-Met inhibitors and were both treated using a multikinase inhibitor. As such, observed responses may be due to effects on targets other than c-Met, including VEGFR2. This hypothesis is supported by recent evidence from our group showing that *TP53* mutations predict responses to anti-VEGFR2 therapies[25]. In addition, these data also suggests that c-Met inhibition may be more clinically efficacious when utilized in a supporting role to block resistance pathways in other targeted agents rather than being utilized alone[1, 2, 4]. These data are preliminary findings that warrant further investigation in a randomized clinical trial setting.

We observed no difference in survival in patients with a *MET* alteration versus those without, although in multivariate analysis patients with *MET* alterations trending towards a worse OS (HR=1.8, p=0.13). However it is possible that separate analyses of *MET* variations and amplifications may yield different results. Due to the limited incidence, we did not observe any significant differences in the characteristics of patients with *MET* alterations. In addition, a number of concomitant mutations were identified in patients harboring *MET* variations, whereas there were none in patients with *MET* amplification other than PTEN loss.

Outside of limitations inherent to all retrospective reviews, the low frequency of *MET* variations and amplifications observed in this study did not provide sufficient statistical power to stringently assess demographic and patient characteristic differences. *MET* variation testing was also done with some heterogeneity. Although the majority of patients were tested at MD Anderson core laboratories, a minority was tested with outside platforms. In addition, mutations and amplifications were often grouped within a single stratum. With regard to amplifications, the relatively low gene copy numbers found in this study (range 2.12-2.78) may be inadequate to exhibit biological differences. Finally, the few number of patients, especially *MET* altered patients, treated on c-Met inhibitor phase I trials makes generalizing these observations into a larger patient populations difficult. However, despite these limitations, to our knowledge this is one of the first studies to substantially compare the demographic and clinical characteristics of ovarian cancer patients with *MET* altered patients and their response to treatment with c-Met inhibitors. The findings of this analysis provide insight into the clinical characteristics associated with *MET* alterations and, if validated in the prospective setting, may lead to improved strategies utilizing c-Met therapies in this prognostically poor patient population.

## METHODS

### Patients

We retrospectively reviewed the medical records of all consecutive patients presenting to our Phase I Clinic starting in May 2010 to November 2012 (n=3607). Patients presented for treatment of recurrent or metastatic disease, usually after multiple prior treatments (range 0-13). Eligibility criteria for inclusion in this study were a histologic diagnosis of ovarian, fallopian tube, or primary peritoneal carcinoma and testing for *MET* nucleotide variations and/or amplification (n=178). This study was approved by the MD Anderson Institutional Review Board and patient confidentiality was maintained following Health Insurance Portability and Accountability Act guidelines.

### Tissue samples and molecular analysis

*MET* nucleotide variation and amplification status were tested using archival formalin-fixed, paraffin-embedded tissue blocks in addition to biologic material from fine needle aspiration biopsies or excised primary or metastatic tumors from diagnostic and/or therapeutic procedures. Histology was centrally reviewed in the MD Anderson Department of Pathology. *MET* nucleotide variations were assessed in several Clinical Laboratory Improvement Amendment-certified laboratories using a single test or as part of a gene panel. The majority of patients (n=160) were analyzed at the MD Anderson core laboratories. Of the remaining samples, 15 were tested at Knight Diagnostics (Portland, OR), 2 at the Baylor College of Medicine (Houston, TX), and 1 using a Foundation One platform (Foundation Medicine, Cambridge, MA) as previously described[13].

*MET* amplification status was analyzed via fluorescence in situ hybridization (FISH) following institutional protocols at MD Anderson. Copy numbers were expressed as a gene copy number in relation to *CEP7*, a gene located near the centrosome of the same chromosome. Gene copy numbers of 2-fold or greater were considered amplified.

### Treatment and evaluation

All patients were enrolled when possible in a Phase I clinical trial(s) judged to be clinically appropriate by the patient's attending physician. Because confidentiality agreements with the providing pharmaceutical company stipulate that c-Met inhibitor identities cannot be disclosed, c-Met agents were classified as being in one of three categories: c-Met-specific inhibitor (3 separate trials), c-Met inhibitor with multikinase activity (2 separate trials), and c-Met inhibitor with multikinase kinase activity in combination with a VEGFR2 kinase inhibitor (1 trial). All c-Met inhibitors with multikinase activity also targeted the VEGFR2 receptor, a molecule associated with known clinical efficacy in ovarian cancer[14]. Patients were treated until clinical or radiologic disease progression, development of unacceptable toxicities or death, clinical judgment necessitating patient removal, or withdrawal of patient consent.

Clinical assessments were performed according to the specific requirements of individual protocols, typically once prior to the initiation of treatment and then at least every treatment cycle. Treatment responses were primarily assessed using computed tomography scans, magnetic resonance imaging, and/or positron emission tomography conducted prior to therapy and every 2 cycles thereafter (6-8 weeks). Radiographs were read in the Department of Radiology at MD Anderson and reviewed by physicians in our Phase I Clinical Trials Program. Objective responses were determined according to RECSIT 1.0 or 1.1 criteria, as specified by individual protocols, and the best responses achieved were recorded[15].

### Statistical Methods

Patient demographics and tumor molecular and histologic characteristics were summarized in relation to *MET* nucleotide variation and amplification status. Time to treatment failure (TTF) was defined as the time from the initiation of therapy to its cessation for any reason. Categorical and continuous variables, including TTF and stratification by *MET* alteration were compared utilizing Fisher's exact and Wilcoxon rank-sum tests, respectively. Overall survival (OS) was assessed starting from the date of the first appointment in the Phase I Clinic using Kaplan-Meier analysis with comparisons via the partial likelihood ratio test. Multivariate and univariate hazard ratios (HR) were calculated via Cox regression. Wald p-values were reported for multivariate analyses. All tests were two-sided when appropriate and considered significant at *p*<0.05. Statistical analyses were performed using SAS version 9.3 (SAS Institute Inc., Cary, NC).
